# Phonological and syntactic competition effects in spoken word recognition: evidence from corpus-based statistics

**DOI:** 10.1080/23273798.2016.1241886

**Published:** 2016-11-09

**Authors:** Jie Zhuang, Barry J. Devereux

**Affiliations:** ^a^ Brain Imaging and Analysis Center, Duke University, Durham, NC27710, USA; ^b^ Centre for Speech, Language and the Brain, Department of Psychology, University of Cambridge, Cambridge, UK

**Keywords:** Cohort competition, lexico-syntactic competition, subcategorisation frames, corpus data, argument structure

## Abstract

As spoken language unfolds over time the speech input transiently activates multiple candidates at different levels of the system – phonological, lexical, and syntactic – which in turn leads to short-lived between-candidate competition. In an fMRI study, we investigated how different kinds of linguistic competition may be modulated by the presence or absence of a prior context (Tyler 1984; Tyler et al. 2008). We found significant effects of lexico-phonological competition for isolated words, but not for words in short phrases, with high competition yielding greater activation in left inferior frontal gyrus (LIFG) and posterior temporal regions. This suggests that phrasal contexts reduce lexico-phonological competition by eliminating form-class inconsistent cohort candidates. A corpus-derived measure of lexico-syntactic competition was associated with greater activation in LIFG for verbs in phrases, but not for isolated verbs, indicating that lexico-syntactic information is boosted by the phrasal context. Together, these findings indicate that LIFG plays a general role in resolving different kinds of linguistic competition.

## Introduction

Understanding a spoken utterance involves neurocognitive processes at different levels of representation – acoustic, phonological, lexical, semantic, and syntactic. Because speech is necessarily heard sequentially over time, language comprehension is underpinned by continuous processes of resolving transient ambiguities at each of these levels. At both the lexico-phonological and syntactic processing levels, research on whether and how multiple candidate representations are activated and compete for selection has generated several cognitive models (Clifton, Frazier, & Connine, 1984; Ferreira & Clifton, 1986; Luce & Pisoni, 1998; Macdonald, 1992; MacDonald, Pearlmutter, & Seidenberg, 1994; Marslen-Wilson, 1987; Marslen-Wilson & Welsh, 1978; McClelland & Elman, 1986; Trueswell & Tanenhaus, 1994). For example, at the lexical level, the cohort model (Gaskell & Marslen-Wilson, 1997; Marslen-Wilson, 1987; Marslen-Wilson & Tyler, 1980; Marslen-Wilson & Welsh, 1978; Tyler & Marslen-Wilson, 1977) proposes that during spoken word processing, word-initial sounds (e.g. “/æl/” of “alligator”) simultaneously activate a cohort of word candidates (“alcohol”, “albatross”, “alligator”) sharing the same initial sound sequence (“/æl/”). As more of the speech signal unfolds, some members of the cohort become inconsistent with the perceptual input and their level of activation decays. Words in the cohort that continue to be consistent with the speech input continue to increase in activation, until a point is reached when only one word remains consistent with the input (the *uniqueness point*). At the uniqueness point, the correct word can be determined unambiguously and all other competitors decay from the cohort. Contextual information can facilitate the comprehension process by constraining the set of cohort competitors to those that are acceptable given the context – for example, those cohort members that are grammatically appropriate to the context (Marslen-Wilson, 1987; Tyler, 1984; Tyler & Wessels, 1983). Using a gating paradigm, Tyler (1984) showed a higher drop-out rate of word candidates from the initial cohort pool when the word was preceded by a strongly grammatically constraining sentence context (i.e. where the context constrains the following word to a particular grammatical class, such as <noun>) compared to either a weakly constraining context or to words presented in isolation (no sentential context). This suggests that word candidates that are perceptually consistent with available input in the unfolding utterance are continuously evaluated against the current context and drop out of the cohort pool when they are incongruent with grammatical constraints. Similarly, eye tracking data where French participants listened to spoken instructions suggest that a gender-marked article preceding the noun functions to eliminate the early activation of the gender-inconsistent cohort members (Dahan, Swingley, Tanenhaus, & Magnuson, 2000; Magnuson, Tanenhaus, & Aslin, 2008). Studies such as these demonstrate that cohort competition effects during word recognition are subject to the top-down influence of contextual constraints.

Processes of competition and constraint may also be fundamental mechanisms in how the appropriate syntactic structure for a sentence is constructed. As a sentence unfolds, multiple possible syntactic structures will be consistent with the currently available input. For constraint-satisfaction models of sentence processing, many syntactic possibilities are generated but their likelihood is constrained by lexical information (Hagoort, 2005; MacDonald et al., 1994; Marslen-Wilson, 1973; Vosse & Kempen, 2000). Indeed, behavioural and neuroimaging studies suggest that in the presence of unresolved syntactic ambiguities, people are sensitive to the likelihood or preferences of the competing syntactic possibilities, such that more likely alternatives are stronger competitors (e.g. MacDonald et al., 1994; Novais-Santos et al., 2007; Rodd, Longe, Randall, & Tyler, 2010; Shapiro, Nagel, & Levine, 1993; Trueswell & Tanenhaus, 1994; Tyler et al., 2011; Tyler & Marslen-Wilson, 1977). For example, when a verb is encountered, the multiple possible argument structures licenced by the verb vary in their likelihood for the verb, and so constraint-satisfaction models will posit that the more likely argument structures will be the stronger competitors.

However, it is unclear whether syntactic information associated with a verb is automatically activated whenever it is heard. Although such information must be activated in order to understand the word’s syntactic role when it appears as part of a larger utterance, it may not be relevant to recognising the word in isolation. One proposal is that lexico-syntactic information is only triggered when the word appears in a context requiring greater linguistic (i.e. syntactic or morphological) processing (Longe, Randall, Stamatakis, & Tyler, 2007; Tyler, Randall, & Stamatakis, 2008). Evidence for this claim is that written verbs produce greater activation than nouns in left posterior middle temporal gyrus (LpMTG; BA 21) when they are in phrasal contexts (e.g. “you drive”, “a battle”), but not when they occur in isolation (Tyler et al., 2008). Furthermore, verbs in minimal phrasal contexts show greater activation than isolated verbs in left inferior frontal gyrus (LIFG) as well as LpMTG, implicating both of these regions in the processing of verbs’ lexico-syntactic information. In these earlier studies, however, the syntactic information associated with words was investigated at the level of their form-class (i.e. noun or verb). In the present study we aim to test the hypothesis regarding the automaticity of lexico-syntactic processing by investigating the activation of lexico-syntactic knowledge at a more fine-grained level, within the verb class, by modelling the processing implications of verbs’ subcategorisation frame (i.e. argument structure) information. To this end, we use a new corpus-based approach to measuring a particular aspect of verb lexico-syntactic processing, namely activation of and competition between subcategorisation frame possibilities for verbs.

In parallel to the cognitive models, contemporary neural models have revealed a left-lateralised fronto-temporal network underpinning fundamental language functions (e.g. Boatman, 2004; Dronkers, Wilkins, Van Valin, Redfern, & Jaeger, 2004; Hickok, 2012; Hickok & Poeppel, 2004, 2007; Indefrey & Levelt, 2004; Scott & Wise, 2004; Tyler & Marslen-Wilson, 2008). Syntactic representations are mainly supported by the LIFG and left posterior temporal regions in a dynamic network (Papoutsi, Stamatakis, Griffiths, Marslen-Wilson, & Tyler, 2011; Tyler et al., 2011; Tyler & Marslen-Wilson, 2008; Tyler, Cheung, Devereux, & Clarke, 2013), in which LIFG functions to integrate component words into a coherent syntactic structure (Hagoort, 2005, 2013). In terms of cognitive competition processes, the LIFG has long been claimed to play a domain-general role of selection among competing representations (Badre, Poldrack, Paré-Blagoev, Insler, & Wagner, 2005; Miller & Cohen, 2001; Thompson-Schill, Bedny, & Goldberg, 2005; Thompson-Schill, D’Esposito, Aguirre, & Farah, 1997). Supporting evidence has come from consistent activation of LIFG across various selection and competition studies, including lexical competition (Zhuang, Randall, Stamatakis, Marslen-Wilson, & Tyler, 2011; Zhuang, Tyler, Randall, Stamatakis, & Marslen-Wilson, 2014), semantic competition (Rodd, Davis, & Johnsrude, 2005), and sentential syntactic competition (January, Trueswell, & Thompson-Schill, 2008).

To explore the relationship between brain mechanisms for lexico-phonological and lexico-syntactic processing, we performed an fMRI study in which participants listened and made lexical decisions to a series of spoken nouns and verbs, presented either in isolation or preceded by a short grammatical context. In the grammatical context condition, each noun and verb (e.g. “book”, “examine”) was preceded by a function word (e.g. “the”, “you”) which constrains the target word’s form-class to either noun or verb (e.g. “the book”, “you examine”). Compared to words presented in isolation, these minimal phrasal contexts should function to reduce the cohort of word-form competitors of the stems to only those candidates which are form-class consistent with the phrasal context, as found in behavioural studies (Dahan et al., 2000; Tyler & Wessels, 1983). Any cohort competition effect found in the isolated word condition should be attenuated when isolated nouns and verbs occur in constraining contexts, because in these contexts a smaller number of grammatically congruent word candidates remain in the cohort. For syntactic competition, however, we predict a reverse activation pattern – a greater syntactic competition effect for verbs presented in phrases, where the phrasal context triggers the activation of competing argument structure possibilities for the verb, and a reduced syntactic competition effect for verbs presented in isolation, where verb lexico-syntactic knowledge is not relevant.

Investigating lexico-phonological and syntactic processing through the prism of competition necessitates measures of both lexical competition over cohort members and syntactic competition over the parse possibilities consistent with a given utterance. For our stimuli, lexico-phonological competition is measured by “cohort size” – the number of word candidates in a cohort that share the same initial two phonemes with the target word (e.g. “/æl/” of “alligator”) (Tyler, Voice, & Moss, 2000; Zhuang et al., 2011). Lexico-syntactic competition is competition over the different subcategorisation frame (i.e. argument structure) possibilities that are consistent with the verb. In cognitive psychology and cognitive neuroscience studies, the likelihood of different subcategorisation frames for verbs has typically been estimated from behavioural experiments, where, for example, participants write down continuations to incomplete sentences or rate sentences for naturalness (e.g. Connine, Ferreira, Jones, Clifton, & Frazier, 1984; Garnsey, Pearlmutter, Myers, & Lotocky, 1997; Novais-Santos et al., 2007; Shapiro et al., 1993). However, some studies suggest that the resultant behavioural frequencies may not provide an accurate reflection of the true frequency of occurrence of each subcategorisation frame with a given verb in corpus data (Merlo, 1994; Roland & Jurafsky, 1998). In particular, the probability that a participant chooses a particular frame for a verb may not reflect the frequency with which that frame is used with the verb in natural language. On the assumption that argument structure expectations associated with verbs reflect statistical data on people’s *experience* of verbs in the language (Garnsey et al., 1997; Lapata, Keller, & Walde, 2001; Merlo, 1994), we utilised a novel approach to describing verb lexico-syntactic knowledge, where competition between different subcategorisation frame possibilities for a verb is measured by the entropy of the conditional probability distribution of subcategorisation frames given the verb, estimated from corpus data. When the competing possibilities are equally likely, the entropy of the distribution will be high, and when one competitor is significantly more likely than the others, entropy will be low. If, as we predict, phrasal context triggers lexico-syntactic processing, then verbs with greater syntactic competition as measured by entropy should show greater activation in regions involved in competition and selection processes, such as LIFG. In contrast, for verbs in isolation we predict a reduced or null syntactic competition effect. Our predictions therefore posit a double-dissociation pattern for lexico-phonological and lexico-syntactic competition in the isolated words and phrases conditions.

## Methods

### Participants

Sixteen healthy volunteers (5 males, 11 females, aged 18–34 years) took part in this study. All were native speakers of British English with normal hearing and were right-handed (Edinburgh Handedness Inventory; Oldfield, 1971). They gave informed consent and were compensated for their time. This study was approved by the Cambridgeshire 3 Research Ethics Committee, UK.

### Design and materials

We first selected a set of 160 nouns and 160 verbs from the CELEX database (Baayen, Piepenbrock, & van Rijn, 1993). The nouns and verbs were matched in imageability, frequency, number of syllables, and number of phonemes. All words were form-class-unambiguous, meaning they could only occur as either a noun or verb, as determined by their frequencies in CELEX. We obtained measures of lexico-phonological competition for each noun and verb stem using *cohort size*, that is, the total number of words sharing the first two phonemes with the target word (e.g. “/æl/” of “alligator”) (Tyler et al., 2000; Zhuang et al., 2011). Lexico-phonological competition increases when cohort size becomes larger with more word candidates competing with each other in the cohort.

**Figure 1.**
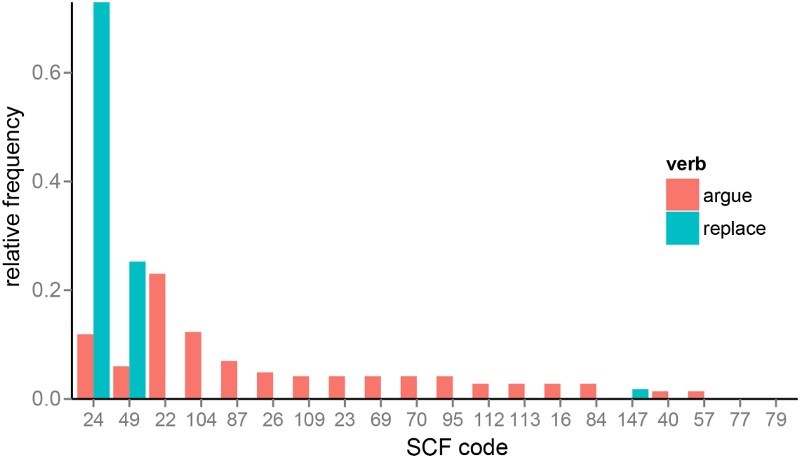
Relative frequency distribution across subcategorization frames (SCFs) for a low entropy verb (“replace”) and a high entropy verb (“argue”). SCF codes refer to the subcategorisation frame classification scheme used in VALEX (Korhonen et al., 2006). “Replace” occurs with just two frames over 98% of the time; with the NP complement frame (SCF 24, e.g. “He replaced the door”) and with the NP-PP frame (SCF 49, e.g. “He replaced the gold with silver”). “Argue” has high entropy because it occurs with many different frames; as well as the NP and NP-PP frames it also occurs with the intransitive frame (SCF 22; “They argued”), the sentential complement frame (SCF 104; “He argued that it was wrong”), the PP frame (SCF 87, “He argued with me”), and so on.

For verbs only, we also manipulated syntactic competition since each verb can be used with a variety of subcategorisation frame possibilities, which differ in their relative likelihood for the verb. Using VALEX, a lexicon of verb subcategorisation frame behaviour derived from large corpora (Korhonen, Krymolowski, & Briscoe, 2006), we obtained the relative frequency with which each verb was used with the 163 subcategorisation frames specified by VALEX. We adopted an information-theoretic framework for measuring competition between subcategorisation frames, in which the notion of competition corresponds to the uncertainty or unpredictability associated with a verbs’ relative frequency distribution over the frames. Specifically, we measured syntactic competition for verbs as the entropy over subcategorisation frame relative frequency distributions (see e.g. Moscoso del Prado Martin, Kostic, & Baayen, 2004; Tabak, Schreuder, & Baayen, 2005, for a similar approach to calculating uncertainty over inflection possibilities). For verbs that tend to have a few highly preferred frames (e.g. *replace*, with transitive frames) the entropy of the subcategorisation frame distribution will be very low, whereas for verbs that tend to be used equally often with many different frames (e.g. *argue*), the entropy of the subcategorisation frame distribution will be high (Figure 1).

On the hypothesis that the activation of and competition between different subcategorisation frame options is an important factor in verb processing, we would expect that a verb that primarily occurs with a single frame and only rarely occurs with other frames differs from a verb that occurs with many frames with similar frequency, even if the verbs have the same number of subcategorisation frame options. Furthermore, by utilising a measure of verbs’ syntactic behaviour derived from corpora, we avoid having to investigate syntactic processing effects through the use of syntactically or semantically anomalous conditions, such as designs with grammatical errors, violations of verb argument structure (Raettig, Frisch, Friederici, & Kotz, 2010), or implausible interpretations (Price, 2010). The entropy measure gives a continuous measure of syntactic competition, which was matched across the high and low cohort competition conditions of the experiment.

Words were presented both as isolated stems and in phrases. For the phrasal condition, each word was preceded by a function word which constrained its form-class to either a noun or verb (e.g. “a monkey”, “you examine”). Three articles (“a”, “the”, and “this”) and three pronouns (“I”, “they”, and “you”) were used as preceding contexts for the nouns and verbs in the phrase conditions, respectively. To avoid repetition of the same stem words across the isolated word condition and the phrase condition for any participant, the stimuli within each condition were divided into matched halves to form two experimental versions. Each participant only received one of the two versions, such that each participant heard all 320 nouns and verbs only once, half in the isolated stem condition and half in the phrase condition.

Our use of a single one-syllable determiner or pronoun as the grammatical context in the phrase condition assumes that even such a minimal linguistic context is sufficient to trigger automatic syntactic processing of the verb (in particular, activation of verb subcategorisation frame possibilities) that is absent in the isolated word condition. Although a richer lexico-syntactic context (i.e. if the verb was embedded in a complete sentence) may make detailed lexico-syntactic processing of the verb more likely and even obligatory, a richer context would also introduce acoustic, semantic and syntactic structure variability that is irrelevant to our experimental questions and which would not be present in the isolated word condition. Previous research (Tyler et al., 2008) has shown that a minimal context is sufficient to generate increased activation for verbs relative to nouns and relative to verbs in isolation.

We used a lexical decision task and included an equal number (320) of non-words as fillers, half of which were in the isolated word condition and half in the phrase condition with the same preceding pronouns and articles. We also included two baseline conditions, one of which was composed of 80 null events (silence), and the other of 280 items of envelope-shaped musical rain (MuR). Similar to speech, the MuR items contain a complex and rapidly changing frequency composition (Uppenkamp, Johnsrude, Norris, Marslen-Wilson, & Patterson, 2006). MuR can be used to separate low-level acoustic processing from lexical processing, as it activates the primary auditory receiving areas (Heschl’s gyrus and planum temporale) to a similar level as speech, but produces less activation than speech in secondary auditory regions such as the anterior and superior STS/STG (Scott, Blank, Rosen, & Wise, 2000; Tyler et al., 2010). One hundred and sixty of the MuR stimuli had normal pitch while the other 120 were made low-pitched by reducing the pitch of the original MuR items. This contrast enabled us to use a high/low judgement of pitch task with the MuR stimuli, which was chosen to approximate the attentional demands of the lexical decision task that was used with the words and non-words. Half of the MuR items were matched for duration to the real words, and the other half was matched for duration to phrases. Non-word and baseline items were the same across the two experimental versions.

Noun and verb phrases were naturally longer (on average by 198 ms) than stems due to the presence of the function word. To account for this, duration was partialled out as an extraneous covariate in the analyses on both the behavioural and imaging data.

### Procedure

The stimuli were recorded by a female native speaker of British English at a sampling rate of 44,100 Hz and downsampled (22,050 Hz, 16 bit resolution, Mono-channel) for presentation with the experimental software. To make the stimulus sounds as natural as possible, the words and non-words were recorded once in isolation and once in phrases, rather than splicing the isolated words from the recordings of the phrases. The mean duration was 628 ms (SD = 113 ms) for the isolated words and 833 ms (SD = 123 ms) for the phrases.

The experiment consisted of two versions. In each version half of the stimuli were presented as isolated words, and half as phrases. Half of the participants were randomly allocated to each version. Each experimental version was composed of 1000 items: 320 real words (160 stem words, 160 phrases), 320 non-words, 280 musical rain items and 80 null events. The nonword filler and baseline items were randomly interspersed with the real word stimuli with the same order across the two versions.

Spoken stimuli were delivered to participants during scanning via the high-quality Nordic Neuro Labs (NNL) electrostatic headphones. Participants were instructed to make a lexical decision to real words and non-words in isolation or in phrases (i.e. the second word in the phrases) by pressing response keys using their right hand index finger for real words and middle finger for non-words, and also to the pitch of MuR items (normal pitch MuR or low-pitch MuR with index and middle fingers, respectively). No task was required on the silence trials. Participants were asked to respond as quickly and accurately as possible on each trial. Response times were recorded from the beginning of the target word for both words in isolation and in phrases.

### MRI acquisition and imaging analysis

Scanning was performed on a 3 T Tim Trio (Siemens, Munich, Germany) at the MRC Cognition and Brain Sciences Unit, Cambridge, UK, using a gradient-echo EPI sequence with head coils. Each functional scan consisted of 32 oblique axial slices, 3 mm thick (0.75 mm gap between slices) with in-plane resolution of 3 mm. Repetition time (TR) = 3.4 s, acquisition time (TA) = 2 s, echo time (TE) = 30 ms, flip angle 78°, and field of view = 192 mm × 192 mm. MPRAGE T1-weighted scans were acquired for anatomical localisation.

We used a fast sparse imaging protocol (Hall et al., 1999) in which speech sounds were presented in the 1.4 seconds of silence between scans. There was a silent gap of 100 ms between the end of a scan and the onset of the subsequent stimulus, minimising the influence of preceding scanning noise on the perception of the speech sounds, especially their onsets. The time between successive stimuli was naturally jittered under this protocol since the duration of each stimulus varies, which increases the chance of sampling the peak of the haemodynamic response.

Pre-processing and statistical analysis were carried out in SPM5 (Wellcome Institute of Cognitive Neurology, London, UK. www.fil.ion.ucl.ac.uk), under MATLAB (Mathworks Inc., Sherborn, MA, USA). EPI images were realigned to the first EPI image (excluding five initial lead-in images) to correct for head motion, then spatially normalised to a standard MNI (Montreal Neurological Institute) EPI template, using a cutoff of 25 mm for the discrete cosine transform functions. Statistical modelling was done in the context of the general linear model as implemented in SPM5, using a 8 mm full-width half-maximal Gaussian smoothing kernel.

In the fixed effect analysis for each participant, we used a parametric modulation design (Büchel, Wise, Mummery, Poline, & Friston, 1996; Henson, 2003) to model the experimental conditions. Two analyses were performed with the first model focussing on phrasal context effect and cohort competition (cohort size) effect over nouns and verbs, and the second model on lexico-syntactic competition (entropy) effect for the verbs (the two analyses reflect the fact that cohort size is defined for both nouns and verbs but subcategorisation frame entropy is defined only for the verbs). In the first analysis, the design matrix of each scanning session consisted of five independent events (isolated words, word phrases, non-words, MuR, and null events) with the first two events (isolated words, word phrases) modulated by three parametric modulators: stimulus duration, word frequency, and cohort size (log-transformed). All three modulators were composed of continuous data, and they were orthogonolised in a serial order from left to right, so the duration and frequency difference among items was partialled out for the effect of cohort competition (as is standard for SPM parametric modulator analysis). In the second analysis, the design matrix of each scanning session consisted of six independent events (verbs in isolation, verb phrases, nouns, non-words, MuR, and null events) with the first two events (verbs in isolation, verb phrases) modulated by three parametric modulators: stimulus duration, word frequency, and syntactic competition (entropy). As in the first analysis, all modulators were composed of continuous data, and they were orthogonolised in the same serial order (stimulus duration and word frequency were partialled out for the effect of syntactic competition).

In addition to the syntactic competition effect, another approach to investigating the neural basis of syntactic representation is to compare nouns and verbs. Verbs substantially differ from nouns in that they serve a key grammatical function in sentences by combining other words in a sentence into a coherent representation. Because of the rich grammatical information in verbs (such as information about licenced and preferred argument structures) which is absent in nouns, comparing nouns and verbs provides an additional means of exploring the neural substrate of syntactic processing. An additional analysis was therefore performed to contrast nouns and verbs. The design matrix of each scanning session was composed of four independent events (all words, non-words, MuR, and null events) with the first event modulated by six modulators: stimulus duration, word frequency, nouns in isolation, verbs in isolation, noun phrases, and verb phrases. All the modulators were treated in parallel, with each modulator orthogonolised against all other modulators. For each of the four experimental modulators, stimulus duration and word frequency were partialled out as extraneous variables.

Trials were modelled using a canonical haemodynamic response function, and the onset of each stimulus was taken as the onset of the trial in the SPM analysis model. For word phrases, we took the beginning of the function words as trial onset since the mean duration of the function words was only 215 ms and BOLD signals are insensitive to such short time differences. The data for each participant were first analysed using the fixed effects model and then combined into a group random effects analysis. Activations were thresholded at *p* < .005, uncorrected, at the voxel level, and significant clusters are reported only when they survive *p* < .05, cluster-level corrected for multiple comparisons, unless otherwise stated. SPM coordinates are reported in MNI space. Regions were identified by using the AAL atlas (Tzourio-Mazoyer et al., 2002) and Brodmann templates as implemented in MRIcron (http://www.MRicro.com/MRicron).

Since this study was aimed at investigating modulation within the language processing network, a bilateral fronto-temporo-parietal mask was applied to all the contrasts in the group random effects analyses (Bozic, Tyler, Su, Wingfield, & Marslen-Wilson, 2013; Diaz et al., 2014; Zhuang et al., 2011). This mask consisted of bilateral IFG (BA 44, 45, 47), insula, anterior cingulate, STG, MTG, angular gyri, inferior parietal lobule, and supramarginal gyri. The mask covered the typical left perisylvian language areas that are of theoretical interest and their right hemisphere homologues (Dronkers et al., 2004; Friederici, 2012; Hickok & Poeppel, 2004, 2007; Petersson & Hagoort, 2012; Tyler & Marslen-Wilson, 2008), while excluding regions not typically involved in language processing. For visualisation, the volumetric statistically thresholded activation maps were projected onto the PALS-B12 surface atlas in CARET version 5.6.

## Results

### Behavioural results

The data from two stimulus items (“hurtle”, “you renege”) were removed due to high error rate (100%). Only data from correct responses (93.6%) were included in the reaction time analyses. The reaction times were inverse-transformed to account for the outliers in the data (Ratcliff, 1993; Ulrich & Miller, 1994), then analysed across items (F2). Error analyses are not reported as the overall error rate is quite low (5.4%), and not more than 9.0% in any of the experimental conditions.

As duration was significantly correlated with RTs (*r* = 0.29, *p* < .001), we partialled it out as a covariate in the analyses. In an ANCOVA on all items with duration partialled out, there was a significant effect of phrasal context, *F*_2_(1, 633) = 7.37, *p* < .01, with word phrases (mean RT = 891 ms) being recognised faster (16 ms) than words in isolation (mean RT = 907 ms), indicating a facilitatory effect of grammatical context. Cohort size (log-transformed) significantly correlated with RTs for words in isolation, *r* = 0.12, *p* < .05, but not for word phrases, *r* = −0.001, *p* > .1. To test whether the correlation between cohort size and RT for words in isolation differed from the correlation between cohort size and RT for words in phrases, we performed a Steiger’s *Z* test for non-independent correlations (Steiger, 1980; www.psychmike.com/dependent_correlations.php), and observed a significant difference (*Z* = 2.09, *p* = .036), indicating that increasing cohort competition inhibited the recognition process of words in isolation, but not words in phrases. In other words, phrasal contexts facilitate spoken word recognition by eliminating form-class inappropriate candidates and in doing so attenuate the influence of cohort competition. The correlation between RTs and syntactic competition (entropy) for verbs in isolation was not significant, *r* = −.05, *p* > .1, nor was it significant for verb phrases, *r* = −0.06, *p* > .1. The absence of syntactic competition effects indicate that the behavioural data collected for the lexical decision task during scanning may not be sensitive to lexico-syntactic information.

### Imaging results

We first established that the task and stimuli elicited activation within those regions of the brain typically activated in language tasks by directly contrasting the activation resulting from MuR items (compared to silence) and real words (including all isolated words and phrases, compared to MuR). The contrast of MuR minus silence tapped into the neural basis of low-level acoustic processing, whilst words minus MuR examined the fundamental features of the spoken language processing network over and above that due to sound processing. Greater neural activation for MuR items compared to silence was focussed primarily in the bilateral STG (BA 41, 42, 22), extending into bilateral MTG (BA 22, 21), inferior parietal lobule and supramarginal gyri (BA 40) and LIFG (BA 45). These results replicate previous studies showing that the purely acoustic features of speech-like sounds (as represented by MuR) are processed mainly in bilateral STG around Heschl’s gyrus. Words were associated with significantly greater activation than MuR primarily along bilateral MTG (BA 21, 22), extending into bilateral anterior STG (BA 22, 38) and LIFG (BA 44, 45, 47). In accordance with previous studies (e.g. Davis & Johnsrude, 2003), these two effects show a hierarchical activation pattern with low-level acoustic information analysed in bilateral Heschl’s gyri and surrounding STG, while more inferior and anterior regions (e.g. bilateral MTG, LIFG) are involved in higher level lexical processing.

**Figure 2.**
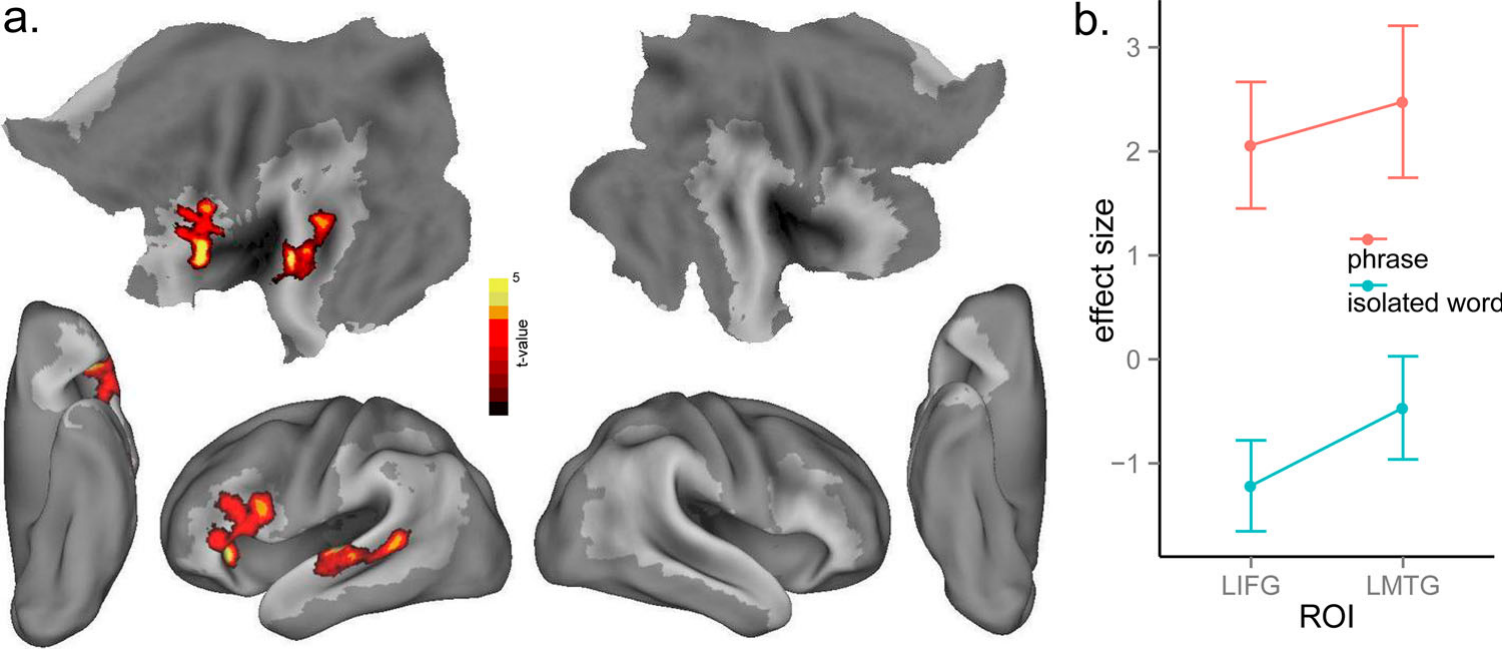
(a) Significant activation for the contrast of word phrases minus isolated words at *p* < .005, voxel-level uncorrected, and *p* < .05, cluster-level corrected. Shaded areas indicate the extent of the language mask. (b) Plot of the mean activation of isolated words and phrases conditions in the significant clusters of (a). Error bars are standard error across subjects.

To investigate the effect of phrasal context, we subtracted words in isolation from word phrases, while partialling out stimulus duration as an extraneous variable. Word phrases elicited more activation than isolated words in LIFG (BA 44, 45, 47), and left middle and posterior MTG (BA 21, 22) (Table 1 and Figure 2(a)). The IFG activation is consistent with previous findings on syntactic processing (e.g. Caplan, Alpert, Waters, & Olivieri, 2000; Hagoort, 2005; Santi & Grodzinsky, 2010; Thothathiri, Kim, Trueswell, & Thompson-Schill, 2012; Tyler et al., 2010, 2011). To visualise the phrasal context effect, we took the significant clusters (Figure 2(a)) as ROIs (regions of interest) using MarsBaR (http://marsbar.sourceforge.net/), and extracted the mean value of activation within the ROIs for words in isolation and word phrases separately (Figure 2(b)).

**Table 1.** Areas of activity for phrasal syntactic effect, cohort competition effect in isolated words, and entropy effect in verb phrases.

		Cluster-level		Voxel-level Pcor		Coordinates		
Regions		Pcor	Extent		*Z*	*x*	*y*	*z*
*Word phrases minus isolated words*								
**LIFG**	**44, 45, 47**	**<0**.**001**	**1270**	**0**.**059**	**4**.**5**	−**34**	**34**	−**2**
				0.812	3.42	−50	12	12
				0.859	3.36	−40	22	20
**LMTG**	**21, 22**	**0**.**001**	**654**	**0**.**113**	**4**.**3**	−**58**	−**20**	−**2**
				0.385	3.86	−48	−40	2
				0.744	3.5	−44	-46	10
*Cohort competition effect in isolated words*								
**L pMTG, pSTG, SMG, IPL**	**21, 22, 40**	**<0**.**001**	**1638**	**0**.**162**	**4**.**15**	−**64**	−**48**	**20**
				0.481	3.72	−50	−28	44
				0.515	3.68	−50	−68	10
**R pMTG, pSTG**	**21, 22**	**<0**.**001**	**1172**	**0**.**283**	**3**.**95**	**50**	−**52**	**18**
				0.544	3.66	46	−46	12
				0.547	3.65	46	−36	14
**LIFG**	**45**	**0**.**026**	**399**	**0**.**288**	**3**.**94**	−**44**	**24**	**4**
				0.784	3.41	−42	22	12
				0.881	3.28	−46	14	2
*Interaction: cohort competition effect in isolated words minus that in phrases*								
**LIFG**	**44, 45**	**0**.**005**	**567**	**0**.**216**	**4**.**05**	**−50**	**18**	**8**
				0.596	3.61	−44	22	4
				0.785	3.41	−58	12	16
**L pMTG, pSTG, SMG, AG**	**21, 22, 39**	**0**.**003**	**638**	**0**.**807**	**3**.**38**	**−48**	**−56**	**16**
				0.927	3.19	−62	−50	24
				0.962	3.1	−54	−50	30
**R pMTG, pSTG, SMG, AG**	**21, 22, 39**	**0**.**002**	**667**	**0**.**825**	**3**.**36**	**46**	**−50**	**16**
				0.843	3.34	48	−58	22
				0.896	3.25	44	−42	12
*Positive correlation from isolated nouns to isolated verbs and verb phrases*								
**LIFG**	**44, 45, 47**	**<0**.**001**	**1673**	**0**.**01**	**4**.**64**	**−42**	**28**	**2**
				0.025	4.42	−54	18	12
				0.036	4.33	−52	12	18
**L MTG, STG**	**21, 22**	**0**.**01**	**813**	**0**.**022**	**4**.**46**	**−50**	**−44**	**8**
				0.23	3.77	−64	−26	2
*Entropy effect in verb phrases*								
**LIFG**	**44, 45**	**0**.**001**	**733**	**0**.**439**	**3**.**8**	**−38**	**0**	**24**
				0.666	3.58	−46	30	2
				0.924	3.24	−38	34	8

**Figure 3.**
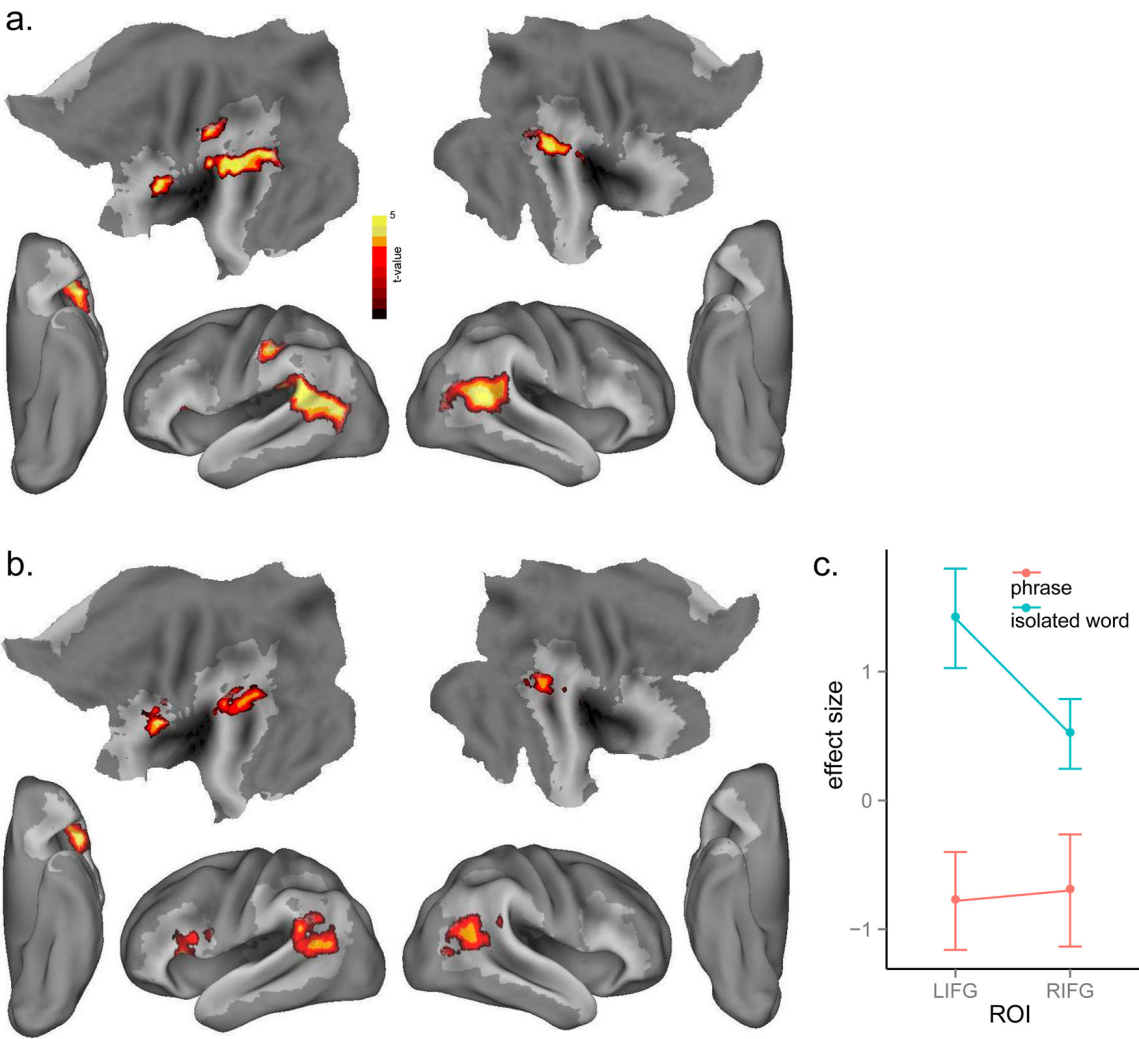
(a) Significant activation for the effect of cohort competition in isolated words at *p* < .005, voxel-level uncorrected, and *p* < .05, cluster-level corrected (b) Significant interaction effect for cohort competition × stimulus type (isolated words *vs.* phrases) (c) Plot of the cohort competition effect in the LIFG and RIFG ROIs from Bozic et al. (2010) across isolated words and phrases.

To examine the brain mechanisms of lexico-phonological competition and whether this competition effect varied as a function of phrasal contexts, we correlated cohort size with neural activation in the isolated word and phrase conditions, separately. For isolated words, increasing cohort competition generated greater activation in the LIFG (BA 45), bilateral posterior MTG/STG (BA 21, 22), extending into left supramarginal gyrus and inferior parietal lobule (BA 40) (Table 1 and Figure 3(a)). However, for the phrases there was no significant effect of cohort competition even at a lower voxel threshold of *p* <.01, uncorrected, and *p* < .05, cluster-level corrected. We performed an interaction analysis by subtracting the cohort competition effect in phrases from the cohort competition effect in isolated words, and observed a significant effect in the LIFG (BA 44, 45) and bilateral posterior regions including posterior MTG/STG, supramarginal gyri and angular gyri (BA 21, 22, 39; see Table 1 and Figure 3(b)).

We further investigated the cohort competition effect using independent ROIs from previous research on lexical competition (Bozic, Tyler, Ives, Randall, & Marslen-Wilson, 2010). We constructed two ROIs based on the competition effect activation peaks ([−48 18 4] and [54 30 0], in LIFG and RIFG) reported by Bozic et al. (2010) with a sphere of radius 10 mm. We then extracted the mean activity of each ROI in the isolated word and phrase conditions, respectively. As shown in Figure 3(c), and consistent with the interaction effect reported above, neural activity for isolated words was significantly higher than that in the phrase condition in the LIFG ROI, *t*(15) = 4.08, *p* < .001, and also in the RIFG ROI, *t*(15) = 2.03, *p* < .05.

**Figure 4.**
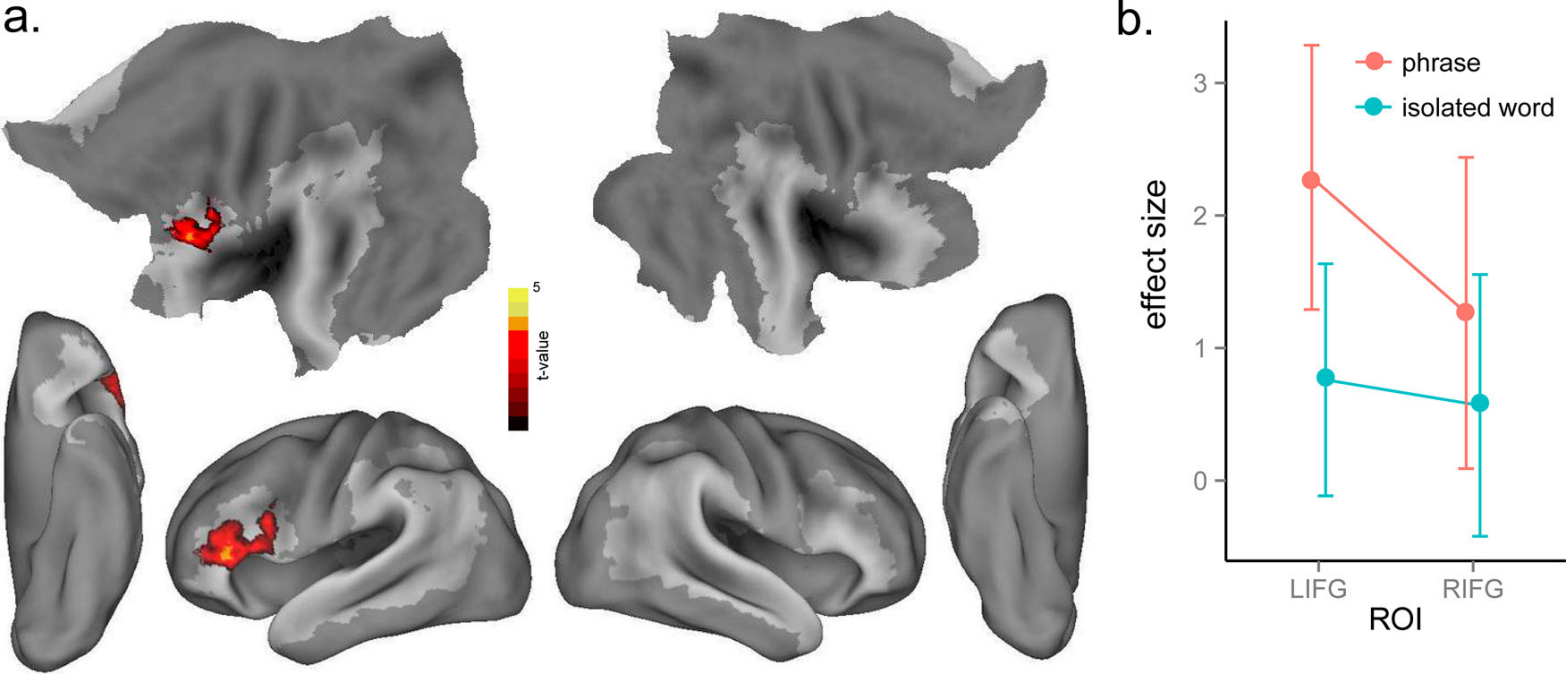
(a) Significant activation for the effect of syntactic competition (entropy) in verb phrases at *p* < .005, voxel-level uncorrected, and *p* < .05, cluster-level corrected. (b) Plot of the syntactic competition effect in the LIFG and RIFG ROIs from Bozic et al. (2010) across isolated verbs and phrases.

As described above, we investigated the neural signatures of lexico-syntactic processing in two ways: by investigating the syntactic competition (entropy) effect within the verbs alone and by contrasting verbs and nouns. We correlated neural activity and syntactic competition for verbs in isolation and phrase conditions, separately, and found no significant effects of syntactic competition for verbs in isolation even when we lowered the threshold to *p* < .01, voxel-level uncorrected, and *p* < .05, cluster-level corrected. In contrast, there was a significant syntactic competition effect for verbs in phrases in LIFG (BA 44, 45) (Table 1 and Figure 4(a)), with greater activation in this frontal region for increasing syntactic competition. However, no clusters survived correction for the whole-brain interaction effect. In the ROI analysis of the interaction effect using the same LIFG and RIFG ROIs as used in the cohort competition analysis, there was greater effect of syntactic competition in the verb phrases than isolated verbs condition in the LIFG ROI, *t*(15) = 1.83, *p* < .05, but not in the RIFG ROI, *t*(15) < 1 (Figure 4(b)). The different entropy effects for the isolated verbs and phrases in the LIFG ROI suggest that activation of lexico-syntactic information associated with verbs is triggered when the word is heard as part of a phrase but not when it is heard in isolation (Tyler et al., 2008).

**Figure 5.**
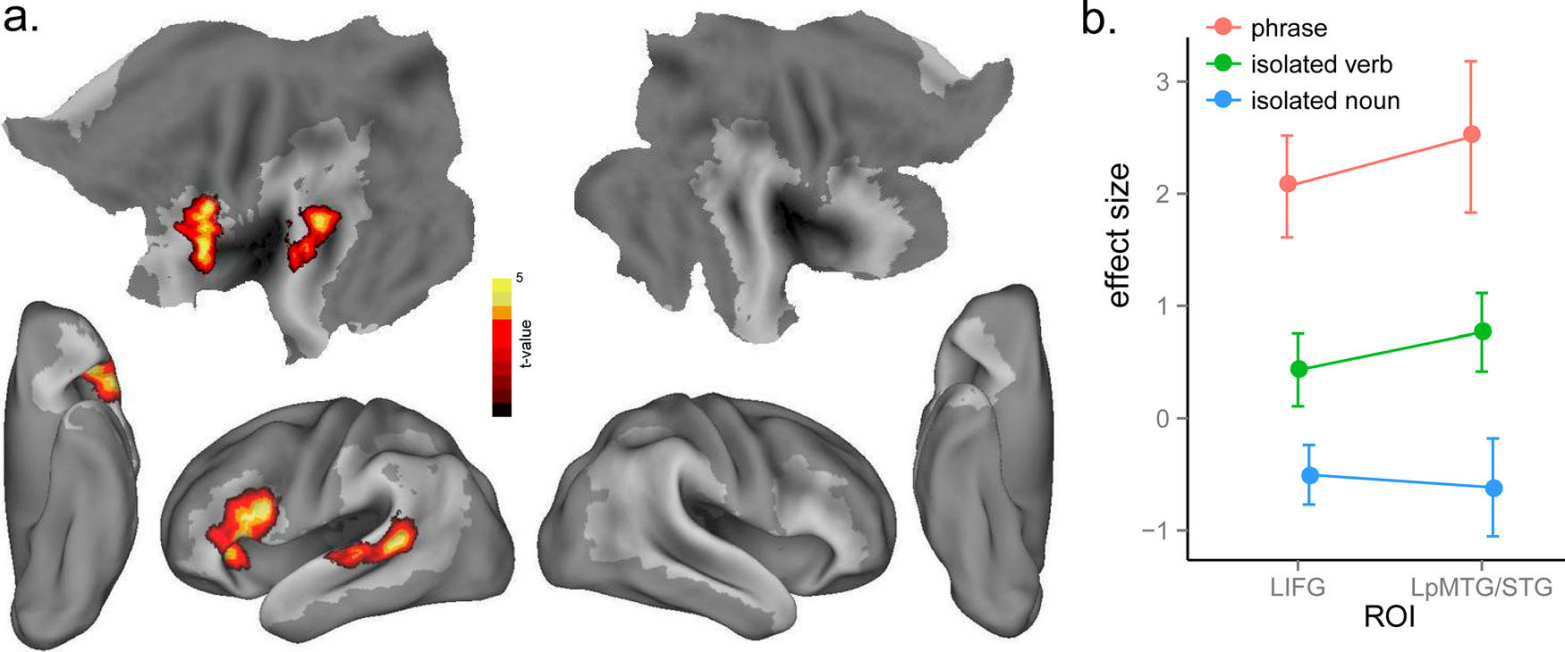
(a) Significant activation for a linear correlation from isolated nouns to isolated verbs and verb phrases at *p* < .005, voxel-level uncorrected, and *p* < .05, cluster-level corrected. (b) Plot of mean activation of each condition in the significant clusters in (a).

In comparing nouns to verbs, no significant differences were found between isolated nouns and isolated verbs, nor between noun phrases and verb phrases, suggesting that no grammatical effect survived in direct comparisons. However, grammatical information might be partially activated for verbs in isolation, showing an intermediate level of activation between isolated nouns and verb phrases. To test this possibility, we performed a correlational analysis with isolated nouns, isolated verbs and verb phrases as three graded levels (1, 2, 3), and found a significant positive correlational effect in the LIFG (BA 44, 45, 47) and left posterior MTG/STG (BA 21, 22) (Table 1 and Figure 5(a)). To visualise the differences between the three levels, mean neural activity in these two significant clusters was extracted in each experimental condition using MarsBaR, then plotted as shown in Figure 5(b). These results demonstrate a linear relationship among these conditions with increasing activation in left fronto-temporal regions from isolated nouns to isolated verbs, to verb phrases.

## Discussion

In this study we investigated how phrasal contexts affect lexico-phonological (measured by cohort size) and lexico-syntactic processing (measured by entropy) in spoken word recognition. We found a significant effect of phrasal syntax with word phrases producing more activation than words in isolation in LIFG (BA 45/44/47) and left middle and posterior MTG (BA 21, 22). There was a significant cohort competition effect in LIFG (BA 45) and bilateral posterior temporal regions for isolated words, but not in word phrases. In the ROI analysis, the cohort competition effects was reduced for phrasal contexts in both LIFG and RIFG ROIs. For lexico-syntactic competition processing, the activation pattern was reversed – there was a significant entropy effect for verbs in phrases, with greater activation in the LIFG ROI (BA 44, 45) for increasing syntactic competition; however, this effect was not significant for verbs in isolation.

The LIFG activation for cohort competition for words in isolation replicates previous findings with the same cohort competition measure (Zhuang et al., 2011). More broadly, this finding accords with previous studies that implicate these frontal regions in resolving competition and selecting among multiple candidates (e.g. Bozic et al., 2010; Moss et al., 2005; Thompson-Schill et al., 2005). The activation in bilateral temporal regions might be related to the extra demands of retrieving lexical phonological and semantic information of more cohort candidates in the high compared to low competition items, since these regions have been claimed to be involved in lexical access of spoken words (e.g. Hickok & Poeppel, 2007; Jung-Beeman, 2005; Longworth, Marslen-Wilson, Randall, & Tyler, 2005). The eliminated cohort competition effect in word phrases is consistent with a role of contextual constraints that function to narrow down the initial cohort size and thus reduce cohort competition. In addition, the onsets of the isolated words may be more salient than the onsets of the same words in phrases due to co-articulation between the nouns/verbs and preceding functional words. The less salient onsets of nouns/verbs in phrases might induce activation of word candidates both within and outside the cohort. The additional activation of non-cohort competitors might have an effect on the level of cohort competition in phrases, but the mechanisms remain unclear since no evidence has shown that non-cohort competitors would prohibit activation of cohort members, or reduce effects of within-cohort competition. There was a significant correlational effect with increasing activation in the LIFG and left posterior MTG/STG when grammatical/syntactic information increased from isolated nouns to isolated verbs and verb phrases. This indicates that grammatical information is automatically activated to a limited extent in verbs in isolation, although it was not detectable in direct comparisons between nouns and verbs. In this study we only chose form-class-unambiguous nouns and verbs as stimuli without any form-class-ambiguous words which could be used as both nouns and verbs in different contexts. The grammatical information is relatively more easily detected in pure verbs without interference from other syntactic properties, compared to ambiguous words (i.e. words that can be either verb or noun, e.g. *kiss*) with multiple possible syntactic roles, only one of which is sensitive to subcategorisation frame information. This might explain why previous research did not find a similar weak grammatical effect in verbs in isolation compared to nouns (e.g. Tyler et al., 2008). One caveat for this correlational effect is that isolated nouns and isolated verbs might also differ from each other in other aspects such as activation levels of action representations, attention, and so on. It is impossible to avoid these potential confounds in analyses with nouns and verbs.

The activation of LIFG (BA 45/44) for syntactic competition for verbs in phrases accords with previous studies that implicate these frontal regions in resolving competition and selecting among multiple candidates (Bozic et al., 2010; Moss et al., 2005; Thompson-Schill et al., 2005; Thothathiri et al., 2012). This effect, together with the null effect of lexico-syntactic competition for verbs in isolation, is also consistent with the notion that neural processing of words is modulated by the extent to which different kinds of relevant (e.g. morphological or syntactic) information is engaged (Longe et al., 2007; Tyler et al., 2008). Lexico-syntactic knowledge of a spoken verb is boosted in a phrasal context because such information is needed to guide phrase structure building for the emerging utterance, whereas it is not necessary to fully activate lexico-syntactic representations for a verb heard in isolation (although it might be automatically activated to a very limited extent) because there is no such need of grammatical integration. Only when multiple subcategorisation frame possibilities are triggered and activated by phrasal contexts could we detect effects of syntactic competition.

The co-activation of left inferior frontal and posterior temporal regions seems to be critical for syntactic representation, both in sentential syntactic processing and in short phrasal syntactic processing (Tyler et al., 2008; Tyler & Marslen-Wilson, 2008). The phrasal syntax effect and the correlational syntactic effect in this study provide further evidence supporting this claim in that even very simple function words (e.g. “you”) could trigger both LIFG and left middle and posterior MTG to work together in building syntactic structures. However, the co-activation pattern of left fronto-temporal regions was not consistently observed in cohort competition and lexico-syntactic competition effects, indicating that the LIFG itself might fulfil the function of selection, with the posterior temporal regions in cohort competition more involved in the retrieval of relevant lexical information.

The cohort competition effect was significant in both LIFG and RIFG in the ROI analysis; however, the syntactic competition effect was significant only in LIFG. This pattern of results suggests that RIFG may be sensitive to competition at a lexico-phonological level, but not at the syntactic level. This is consistent with the dual system model (Bozic et al., 2010; Tyler & Marslen-Wilson, 2008) which posits that a bilateral fronto-temporal system is engaged in basic non-combinatorial processes of lexico-phonological access, whilst a left-lateralised system is specialised for combinatorial linguistic processes, such as syntax.

One notable finding of this study is that the phrasal contexts effect, the correlational syntactic effect, the cohort competition effect for isolated words and entropy effect for verb phrases overlap largely in a sub-region of LIFG – L triangularis (BA 45). L triangularis (BA 45) might be the most frequently reported sub-region of LIFG for multiple cognitive functions, including general control or selection processing (Badre et al., 2005; Bozic & Marslen-Wilson, 2010; Moss et al., 2005; Righi, Blumstein, Mertus, & Worden, 2009; Schnur et al., 2009; Thompson-Schill et al., 1997; Thompson-Schill, Aguirre, Desposito, & Farah, 1999; Wright, Randall, Marslen-Wilson, & Tyler, 2010; Zhuang et al., 2011) and syntactic processing (e.g. Friederici, Bahlmann, Heim, Schubotz, & Anwander, 2006; Kaan & Swaab, 2002; Santi & Grodzinsky, 2007; Tyler et al., 2011). This finding provides direct evidence to support this notion that L triangularis (BA 45) is engaged, at multiple linguistic levels, in selecting an appropriate candidate among competitors and combining several constitutes into a coherent representation.

In summary, this study demonstrates that phrasal contexts function to eliminate cohort competition in recognition of spoken words and boost activation of lexico-syntactic properties within interactive bilateral fronto-temporal processing systems. The LIFG is a critical language region with multiple functions in building syntactic structure, and in resolving lexical and syntactic competition according to different processing needs.
